# Coherent Mid-IR Supercontinuum Generation using Tapered Chalcogenide Step-Index Optical Fiber: Experiment and modelling

**DOI:** 10.1038/s41598-020-59288-6

**Published:** 2020-02-10

**Authors:** Than Singh Saini, Tong Hoang Tuan, Takenobu Suzuki, Yasutake Ohishi

**Affiliations:** Research Center for Advanced Photon Technology, Toyota Technological Institute, 2-12-1, Hisakata, Tempaku, Nagoya 468-8511 Japan

**Keywords:** Optics and photonics, Optical physics

## Abstract

Mid-infrared region of electromagnetic spectrum has increased a lot of scientific and technical interest because of its utility to figure out the molecular fingerprints. Current mid-infrared light sources including quantum cascade lasers, thermal-emitters, and synchrotron radiation are not suitable for various potential applications where we require coherent, portable and broadband light sources. During the current decade, several efforts have been put forwarded to extend the spectral range of the supercontinuum. However, the coherent mid-infrared supercontinuum spectrum in the mid-infrared region has been demonstrated rarely. Here, we demonstrate a coherent mid-infrared supercontinuum using a tapered chalcogenide fiber pumped at various wavelength ranging from 2 µm to 2.6 µm. Experimental observations show that the supercontinuum spectrum extending from ~1.6 µm to 3.7 µm can be achieved using a 3 cm long tapered chalcogenide step-index optical fiber pumped with femtosecond laser pulses at 2.6 µm. To the best of our knowledge, using short pump wavelengths at 2 µm to 2.6 µm in an all-normal dispersion engineered chalcogenide glass fiber, the coherent supercontinuum spectrum has been reported first time. Such coherent broadband light source has its key prominence for the various prospective applications in the fields of bio-medical, sensing, and multiplex coherent anti-Stokes Raman scattering microspectroscopy.

## Introduction

The mid-infrared (mid-IR) supercontinuum generation has attracted a lot of attention in recent years because of the existence of unique absorption bands of most of the molecules in this region^1^. Additionally, mid-IR supercontinuum light sources are expected to have various potential applications including bio-photonic diagnostics, flow cytometry, nonlinear spectroscopy, and infrared imaging^2–5^. Earlier, several optical fiber and waveguide structures have been reported for broadband mid-IR supercontinuum spectrum^6–18^. For lots of the potential applications an intense, coherent, broadband, and compact mid-IR supercontinuum light sources are essential^2,19–21^. Supercontinuum light generated from a photonic crystal fiber was used in a typical single-oscillator multiplex coherent anti-Stokes Raman scattering (CARS) step for the measurement of vibrational bands of cyclohexane sample^22^. A photonic-chip based supercontinuum light source was used in the mid-IR gas spectroscopy for the detection of acetylene (C_2_H_2_) gas^23^. A spatially coherent supercontinuum light source is desirable for high-spatial-resolution imaging. The optical fiber is expected to have promising medium for the design and development of a highly spatially coherent mid-IR light source with the high brightness. Earlier, the broadband mid-IR supercontinuum generation has been reported using the optical fibers in different materials including fluoride, tellurite and chalcogenide, but, its coherence property has not demonstrated extensively.

The coherence characteristic of the supercontinuum spectra generally depends on the geometrical parameters of the fiber, pump conditions, and the spectral broadening mechanism^24^. One of the commonly employed methods to obtained coherent supercontinuum spectra is to pump with femtosecond laser pulses in normal dispersion region. In the fibers offering all-normal dispersion (ANDi) profile, the broadening of supercontinuum spectrum is dominated by the self-phase modulation (SPM) and the optical wave-breaking (OWB)^25^. The optical noise generated by the nonlinear processes (such as modulation instability and the soliton fission) is completely absent in the case of ANDi engineered fibers pumped with femtosecond laser pulses^26^.

Recently, Al-Kadry *et al*. demonstrated broadband supercontinuum spectrum spanning 960 to >2500 nm using an ANDi chalcogenide microwire^27^. Liu *et al*. reported experimentally the coherence mid-IR supercontinuum spectrum expanding from 2.2–3.3 µm can be generated using 2 cm long chalcogenide microstructured optical fiber pumped by femtosecond laser pulses at the wavelength of 2.7 µm^28^. Liu *et al*. numerically examined the coherent mid-IR supercontinuum spectrum in the tapered step-index chalcogenide fibers for various structural parameter and pumping with femtosecond laser pulses of peak power of 1 kW at 4 µm^29^. Li *et al*. demonstrated coherent supercontinuum light extending from 1.4 to 4 µm using a 4 cm long tapered fluorotellurite microstructured optical fiber pumped with femtosecond laser pulses^30^. Zhang *et al*. demonstrated coherent supercontinuum spectrum in an all-normal dispersion engineered Te-based chalcogenide tapered optical fiber pumped at 5.5 µm^31^. Shabahang *et al*. reported supercontinuum spectrum extending from 850 nm to 2.35 µm in a robust chalcogenide glass nanotaper pumped with ps laser at 1.55 µm^32^. Granzow et al. reported an efficient mid-IR supercontinuum generation in an arsenic trisulphide nano-spike waveguide pumped with 65 fs laser pulses at 2 µm^33^. Our group has demonstrated coherent mid-IR supercontinuum generation in step-index tellurite, tapered tellurite, and chalcogenide double clad optical fibers pumped with femtosecond laser system^34–36^. Among all the fibers, the chalcogenide glass fibers are much suitable candidate for mid-IR supercontinuum applications. In the earlier reported results, the coherent supercontinuum spectra in chalcogenide fibers were obtained using the pumping at the longer wavelengths which are not commonly available laser sources. Additionally, at longer wavelengths the chalcogenide glasses offer large two-photon absorption. The pumping at shorter wavelengths considerably decreases the two-photon absorption and sanctions operating in the normal dispersion region. However, the coherent mid-IR supercontinuum spectrum using the chalcogenide fibers pumped at shorter wavelengths (<2.7 µm) has not been demonstrated yet. Very recently, Jain *et al*. numerically reported the M-type fiber structures in chalcogenide and ZBLAN materials for obtaining the zero dispersion wavelength (ZDW) of the core-confined higher-order mode in the spectral range of 2 to 3 µm^37^. Such fibers are expected to have potential application in the future mid-IR supercontinuum sources pumped with established laser pump technology. Sharma *et al*. proposed the design of a triangular-lattice annular-core photonic crystal fiber structure allowing both the steady broadband guided-transmission and the supercontinuum generation of optical vortex beams in fiber^38^. Anashkina *el al*. demonstrated a tapered chalcogenide suspended-core fiber structure for the application of broadband mid-IR wavelength conversion^39^. One of the advantages of the tapering of the fiber is that the length of the fiber for supercontinuum generation can be prominently reduced.

In this work, we have fabricated a chalcogenide tapered fiber with AsSe_2_ as a core and As_2_S_5_ as a cladding glasses. Fabricated fiber exhibits ANDi characteristic upto the wavelength of ~3.95 µm. The coherent mid-IR supercontinuum spectrum is demonstrated using the fabricated ANDi chalcogenide tapered fiber pumped with femtosecond laser system at 2.0 to 2.6 µm (with the step of 0.2 µm). We demonstrate the coherent mid-IR supercontinuum spectrum spanning ~1.6 µm to 3.7 µm using a 3 cm long tapered chalcogenide step-index fiber pumped with femtosecond laser pulses of the peak power of 10.12 kW at 2.6 µm. To justify the experimentally obtained result, a numerical simulation also performed for the same fiber geometrical parameters and the laser pump conditions as they were used in the experiment.

## Chalcogenide Tapered Fiber Design

**Figure 1:**
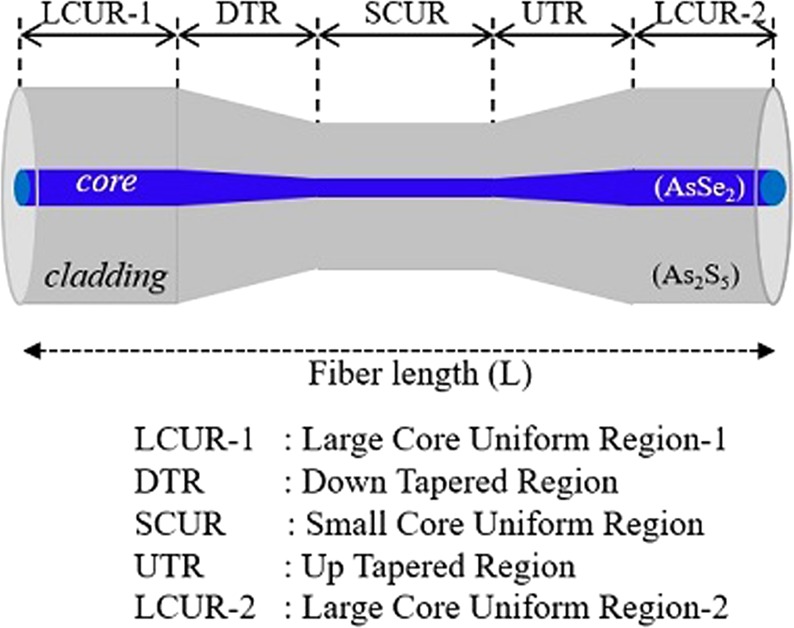
The longitudinal view of the chalcogenide tapered optical fiber structure.

The schematic of the chalcogenide tapered fiber is depicted in Fig. 1. As shown in Fig. 1, the tapered fiber consists of five sections (three uniform, one down-tapered, and one up-tapered) longitudinally. L is the total length of the tapered chalcogenide fiber. The materials of the core and cladding were AsSe_2_ and As_2_S_5_ chalcogenide glasses, respectively. The calculated numerical aperture (NA) and the difference in the refractive indices of the core and cladding were ~1.50 and ~0.5, respectively, at 2.6 µm.

## Fiber Fabrication

**Figure 2:**
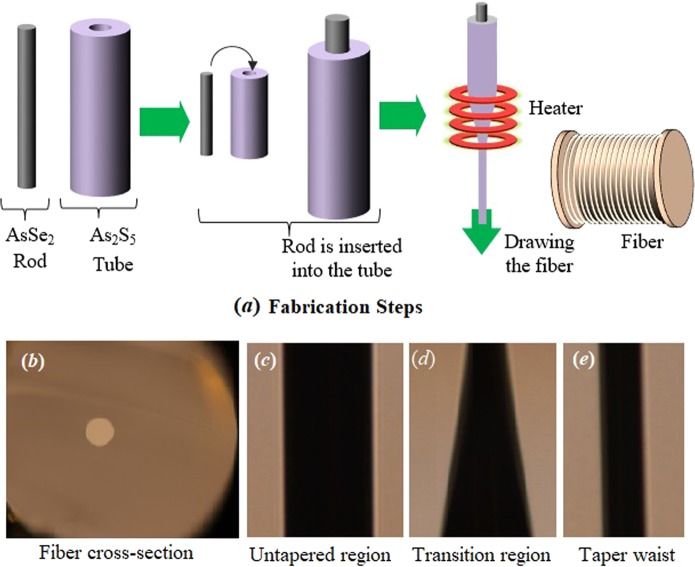
(**a**) The steps in the fabrication process of the chalcogenide step-index optical fiber using rod-in-tube method; (**b**) Cross-section of fabricate fiber; (**c**) Untapered region; (**d**) Transition region; (**e**) Taper waist.

A step-index chalcogenide fiber with AsSe_2_ glass as a core and As_2_S_5_ glass as a cladding was fabricated using the rod-in-tube method. As illustrated in Fig. 2(a), firstly, AsSe_2_ glass rod and As_2_S_5_ glass tube were fabricated by the casting and rotational casting method, respectively. The AsSe_2_ glass rod was elongated and inserted it into the As_2_S_5_ glass tube. Then the combination of the AsSe_2_ rod and As_2_S_5_ tube was elongated simultaneously to obtain the step-index chalcogenide fiber. We adjusted the nitrogen gas pressure as negative to avoid any interstitial hole formation between the core and the cladding during the complete process of fiber drawing. Finally, tapering of the fabricated step-index chalcogenide fiber was performed by employing a home-made fiber tapering system. The bench of the fiber tapering system was allowed to inclined at an angle of 5 degrees from the horizontal position. We set the operating temperature of the circular filament at 180 °C. When the tapering process is done the tapered fiber geometrical parameters were measured using digital imaging system (Nikon: DS-5M-L1) for microscope (Nikon: ECLIPSE ME600L) and illustrated in Fig. 2(b–e). The numerical values of the measured tapered geometrical parameters are provided in Table 1. The core diameter at the ends of the fiber was 15 µm and the minimum core diameter at the tapered waist was measured as 7 µm.

**Table 1:** The geometrical parameters of the fabricated chalcogenide tapered fiber.

Geometrical parameters	LCUR-1	SCUR	DTR/UTR	L
	0.7 cm	3 mm	4 mm	3.0 cm

**Figure 3:**
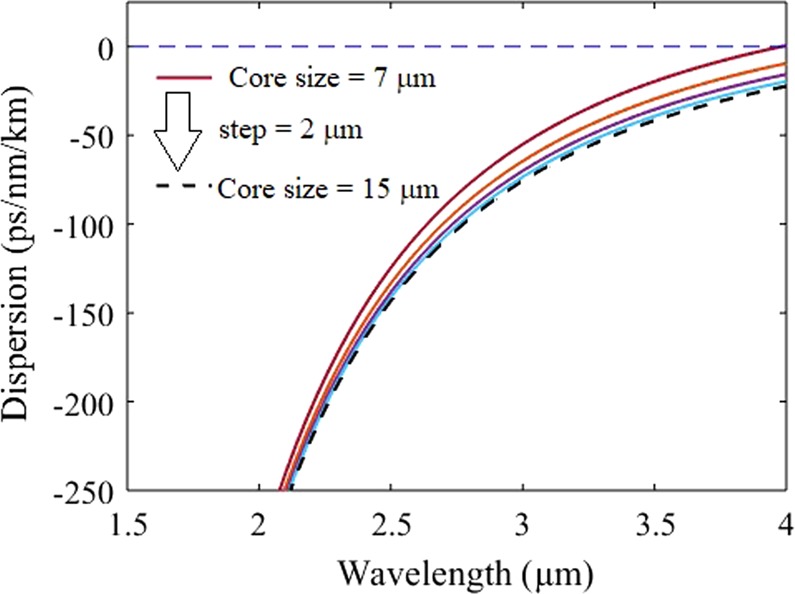
Dispersion profile of the fundamental propagating mode of the fiber with various core sizes varying from 7 µm to 15 µm with the step of 2 µm.

The chromatic dispersion profile of the tapered chalcogenide fiber with core diameter varying from 7 µm to 15 µm is illustrated in Fig. 3. It directs that the chalcogenide fiber offers ANDi characteristic upto the spectral range of 3.95 µm for the fibers with the core size varying from 7 µm to 15 µm. At the wavelength of 2.6 µm, the simulated chromatic dispersion values of the fiber with core diameters of 7 µm and 15 µm are −106.5 ps/nm.km and −125.4 ps/nm.km, respectively.

**Figure 4:**
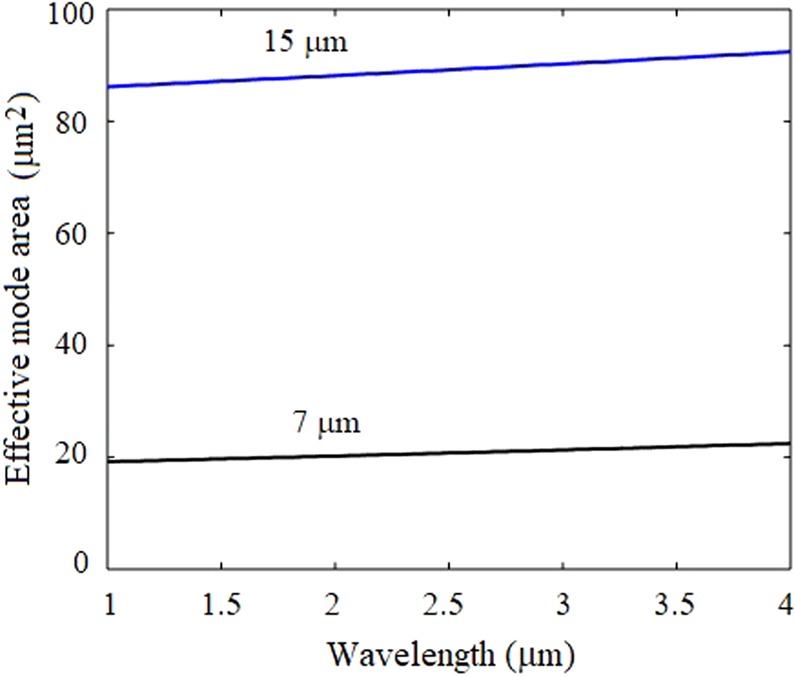
The spectral variations in the effective mode area of the fundamental mode of the fiber with core sizes of 7 µm and 15 µm.

**Figure 5:**
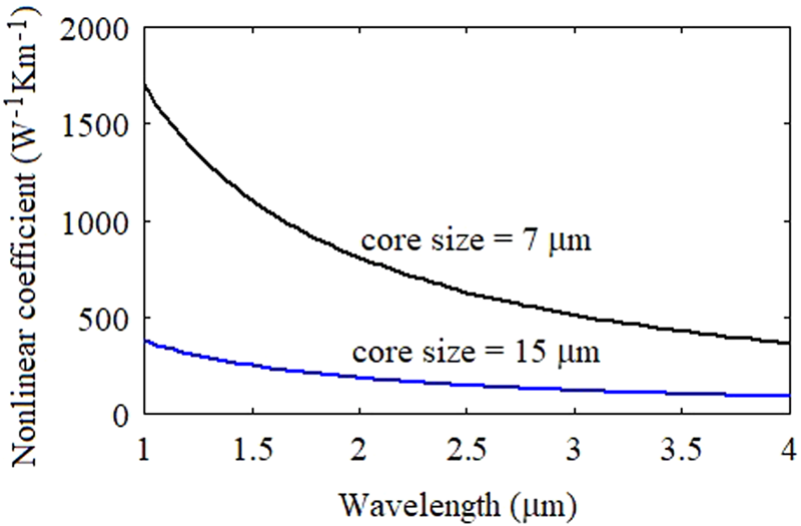
The spectral variations in the nonlinear coefficient of the fundamental mode of the fiber with core sizes of 7 µm and 15 µm.

The spectral variations of the effective mode area of the fundamental mode propagating in the chalcogenide fiber with core sizes of 7 µm and 15 µm is illustrated in Fig. 4. The numerical values of effective mode area of fundamental mode of the fiber with core size of 7 µm and 15 μm are 20.85 μm^2^ and 89.45 μm^2^, respectively, at the wavelength of 2.6 µm. The wavelength dependent nonlinear coefficient of the fundamental mode of chalcogenide fiber is depicted in Fig. 5. The numerical values of nonlinear coefficient of the fiber with core size of 7 µm and 15 µm are 602.7 W^−1^ Km^−1^ and 141 W^−1^ Km^−1^ at 2.6 µm.

**Figure 6:**
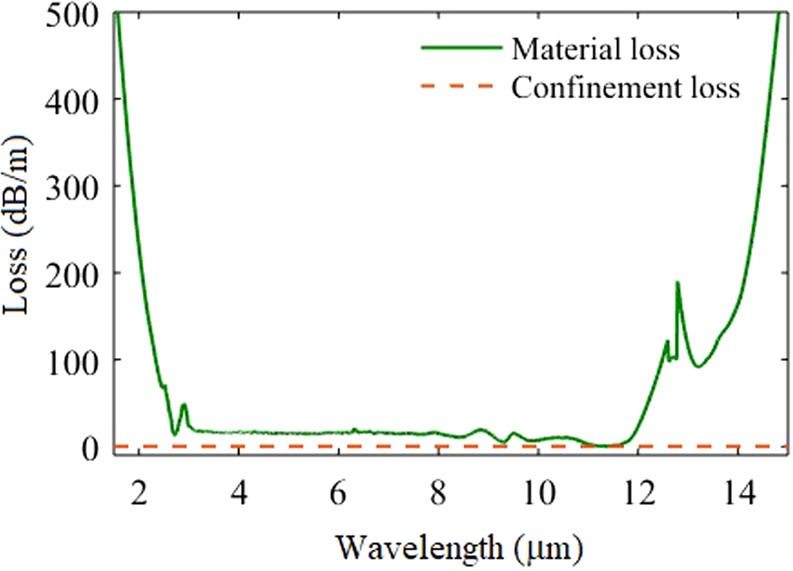
The material loss of AsSe_2_ glass, and the confinement loss of the fiber.

The material loss of AsSe_2_ glass and the confinement loss of the fiber are illustrated in Fig. 6. The absorption peak at ~2.9 µm is due to the OH impurity in the glass, and another absorption peak at ~12.7 µm exists because of Se-OH bonds^40^. The confinement losses of the fundamental mode have been obtained at various core sizes of the fiber varying from 7 µm to 15 μm. It is a well-known fact that the confinement loss increases on decreasing the core size. In Fig. 6, the confinement loss for the fiber with 7 µm core size is provided. As shown in Fig. 6, the confinement loss of the fiber is very less in the whole spectral range even at smallest core diameter at the tapered region (*i.e*. 7 µm).

### Experimental setup

**Figure 7:**
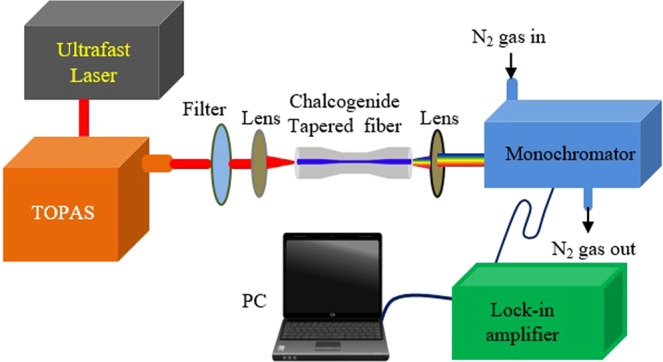
The illustration of the experimental setup used in the measurement of the supercontinuum spectrum from the chalcogenide tapered fiber.

The experimental setup for the measurement of supercontinuum spectra using the fabricated chalcogenide tapered step-index fiber is shown in Fig. 7. An ultrafast Ti-sapphire mode-locked laser (Coherent Mira-900-F) delivering pulses at 800 nm with spectral pulse width of 12 nm was employed as a seed laser source. The seed laser source delivers pulses to the pulse picker regenerative amplifier. The output of the amplifier provides the pulses with the pulse energy of ~1 mJ at the repetition rate of 1000 Hz. Then the amplified laser pulse permits to pass through the traveling wave optical parametric amplifier of superfluorescence (Coherent TOPAS-C) which generates a signal beam of 1.16–1.6 µm and an idler beam tunable from 1.6 to 2.6 µm with the pulse width of 200 fs. A long-pass filter was employed to isolated the signal and idler beams. In our experiment, an idler beam at 2.0 to 2.6 µm wavelengths was allowed to couple into the 3 cm chalcogenide tapered fiber using an aspheric lens (THORLABS, C021TME-D, AR 1.8–3.0 µm) with the focal length of 11 mm. The transmission efficiency of the lens used in the experiment was approximately 60%. The estimated coupled peak power to the fiber was around 10.12 kW at 2.6 µm. The output of the chalcogenide tapered fiber was collected using a ZnSe lens with focal length of 12 mm. The output spectra were measured using a monochromator (Bunkoukeiki CT-25) with 2 nm resolution. The transmission range of the ZnSe lens was 0.6–21.0 µm. The signal received from the monochromator was amplified using a lock-in-amplifier (NF LI5640)^34–36^. Finally, the supercontinuum spectrum was recorded using a computer based spectrometer system.

## Results and Discussion

**Figure 8:**
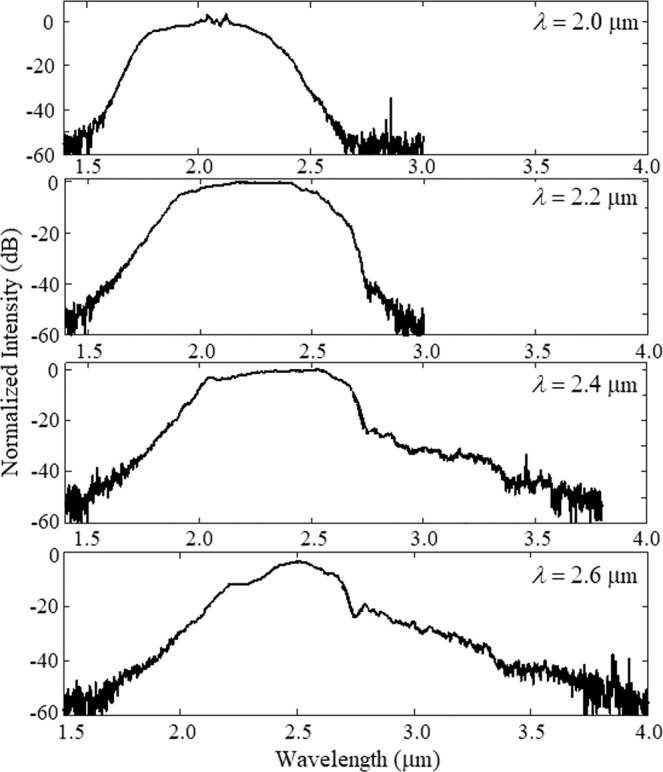
The variations in the broadening of supercontinuum spectra at the output of 3 cm long tapered chalcogenide fiber pumped with different pump wavelengths in the mid-IR region.

In our experiment, a 3 cm long tapered chalcogenide fiber was used to generate supercontinuum spectrum. The measured spectral broadening at the output end of the tapered chalcogenide fiber is shown in Fig. 8 for various pump wavelengths of 2.0 µm, 2.2 µm, 2.4 µm, and 2.6 µm. Figure 8 shows the dependence of the measured supercontinuum spectra from ANDi tapered chalcogenide fiber pumped with 200 fs laser pulses of estimated coupled peak power of 11.88 kW, 11.60 kW, 11 kW, and 10.12 kW at pump wavelengths of 2.0 µm, 2.2 µm, 2.4 µm, and 2.6 µm, respectively. The measured supercontinuum spectral broadening spanned only from 1.5 to 2.6 µm for the pumping at 2.0 µm. When the pumping wavelength increased to 2.6 µm, the long wavelength edge of the supercontinuum spectrum extended and widest supercontinuum spectrum generated. At the pump wavelength of 2.6 µm, the maximum spectral broadening extending from 1.6 µm to 3.7 µm has been measured. For the pumping in normal dispersion regime, initially, SPM is the dominant nonlinear effect in the broadening mechanism of supercontinum generation. Thereafter, OWB and higher order dispersion are responsible for blue and red shifts of the spectrum.

The reported damage threshold of the As_2_Se_3_ glass in terms of laser fluence threshold (F_th_) is 88.4 mJ/cm^2^ for 150 fs laser pulses at 3 µm^41^. In the case of our experimental setup and laser pulse parameters, the maximum allowable peak power should be less than 3.52 MW to avoid the fiber damage. In our actual experiment, the estimated coupled peak power is always less than the laser threshold of the chalcogenide glass. In support of the experimentally obtained result, the superconitnuum spectrum in tapered chalcogenide fiber with the same fiber geometrical parameters and pump conditions has been simulated numerically and discussed in the next section.

### Numerical method and analysis

To compare the measured supercontinuum spectrum, we performed numerical simulation by solving the following generalized nonlinear Schrodinger equation^42^ (see Supplementary Material)

1 $$\frac{\partial \tilde{A}{\prime} }{\partial z}=i\bar{\gamma }(\omega )\exp (\,-\,\widehat{L}(\omega )z) {\mathcal F} \{\bar{A}(z,T){\int }_{-\infty }^{\infty }R(T{\prime} ){|\bar{A}(z,T-T{\prime} )|}^{2}dT{\prime} \}$$

The Eq. (1) was solved by employing the adaptive step size method with the fourth-order-Runge-Kutta algorithm^42^. In the numerical simulations, the geometrical parameters of tapered chalcogenide fiber were taken as given in the Table 1. The material as well as confinement losses of the fiber have been included in all simulations.

The coherence characteristic of the generated supercontinuum spectrum is affected by the existence of the quantum noise of the pulse. We used one-photon-per-mode semi-classical theory to model the noise of the input pulse^43^. The complex degree of coherence was used to consider the deficit in the coherence characteristic of the supercontinuum spectrum due to the spectral phase instability at each wavelength. The relation for the complex degree of coherence is as follows^44^

2 $$|{g}_{12}^{(1)}(\lambda ,{t}_{1}-{t}_{2})|=|\frac{\langle {E}_{1}^{\ast }(\lambda ,{t}_{1}){E}_{2}(\lambda ,{t}_{2})\rangle }{\sqrt{\langle {|{E}_{1}(\lambda ,{t}_{1})|}^{2}\rangle }\sqrt{\langle {|{E}_{2}(\lambda ,{t}_{2})|}^{2}\rangle }}|$$

where *E*_1_ and *E*_2_ are the amplitudes of the electric field for two successive generated spectra. ${g}_{12}^{(1)}=1$ for completely coherent spectrum, and ${g}_{12}^{(1)}=0$ for entirely incoherent light.

**Figure 9:**
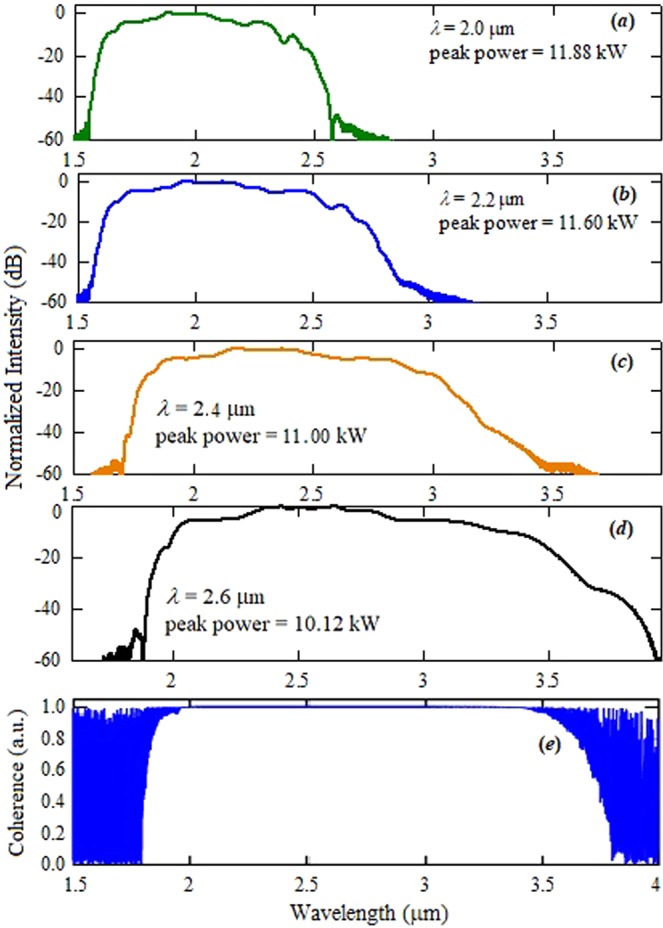
(**a**–**d**) Simulated supercontinuum spectra at the output of 3 cm long tapered chalcogenide fiber pumped at 2.0 to 2.6 µm laser pulse, (**e**) the coherence property of generated supercontinuum spectrum at 2.6 µm.

The simulated spectral broadening of the supercontinuum spectrum from a 3.0 cm long tapered chalcogenide fiber, for the pump wavelengths varying from 2 µm to 2.6 µm, is illustrated in Fig. 9(a–d). In the simulations, the peak power of the pump at various wavelengths has been considered same as it was estimated in the experiment at particular wavelength. The simulated and measured spectral broadening of the supercontinuum spectra are almost similar. In the ANDi fiber, the SPM and the OWB play very important role for the spectral broadening of the supercontinuum spectrum. In the phenomena of SPM and OWB new component of the wavelengths with a phase related to the input pulse are created, and the noise-sensitive soliton dynamics supresses^45^. Therefore, the supercontinuum spectrum maintains the coherence characteristic in the ANDi fibers. As shown in Fig. 9(e), the complex degree of coherence is almost unity (which corresponded to the perfect coherence) within the whole range of the generated supercontinuum spectrum. In the ANDi fiber pumped with ultrafast laser pulses, the nonlinear coupling between nonlinear effects contributes to the suppression of incoherent dynamics and as a result, a highly coherent supercontinuum spectrum is obtained^46^.

**Figure 10:**
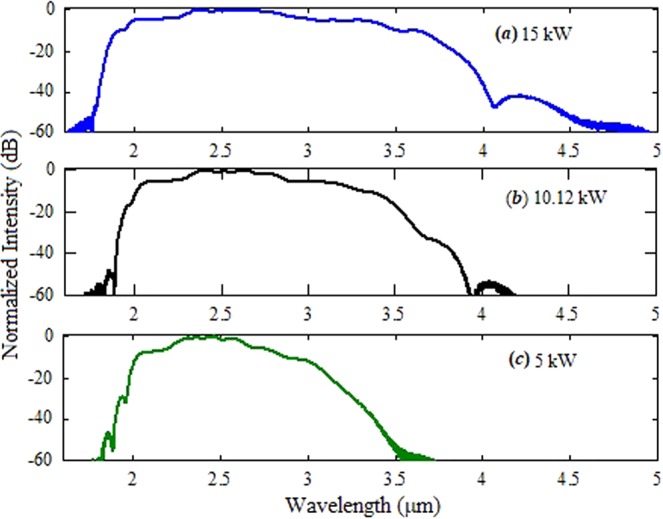
Variations in the supercontinuum spectra when the fiber pumped by the laser pulses of the peak power of (**a**) 15 kW, (**b**) 10.12 kW, and (**c**) 5 kW at 2.6 µm.

The variations in the spectral broadening of the supercontinuum spectrum on laser pulse peak powers are illustrated in Fig. 10. It is clear that when the peak power of input pulse is 15 kW, the supercontinuum spectrum extended upto 4.5 micron at the higher wavelength edge. Therefore, in the experiment, the spectral broadening beyond 3.7 µm is limited by the input power coupled to the core of the fiber.

## Conclusions

In conclusion, we have demonstrated coherent mid-IR supercontinuum generation using a tapered chalcogenide fiber pumped with femtosecond laser pulses at 2.0–2.6 µm. The experimentally measured results show that the coherent broadband supercontinuum spectrum extending from ~1.6 µm to 3.7 µm can be obtained using a 3 cm long tapered chalcogenide step-index fiber pumped with 200 fs laser pulses of the peak power of 10.12 kW and the repetition rate of 1000 Hz at 2.6 µm. We also carried out the numerical simulations to obtain supercontinuum generation in tapered chalcogenide fiber for the same fiber geometrical parameters and pump conditions as they were used in the experiment. The simulated results support the experimentally measured results. In the past, the supercontinuum generation in chalcogenide glasses have been demonstrated with the pumping at higher wavelengths (2.7 µm or more) in the mid-IR region. In this work, the supercontinuum spectrum in chalcogenide fiber pumped with relatively shorter pump wavelengths have been demonstrated for the first time. Such highly coherent mid-IR superontinuum light sources are expected to have potential applications in various fields including early cancer diagnostics, inspecting food quality, gas sensing, high-spatial-resolution imaging, and multiplex coherent anti-Stokes Raman scattering microspectroscopy.

## Supplementary information

Supplementary Information.
