# Supplementary material for: Coherent Mid-IR Supercontinuum Generation using Tapered Chalcogenide Step-Index Optical Fiber: Experiment and modelling
Source: Sci Rep. 2020 Feb 10;10:2236. doi: 10.1038/s41598-020-59288-6 (PMC7010760; doi:10.1038/s41598-020-59288-6)
Supplement: Supplementary file 1 — Supplementary Information. [file 41598_2020_59288_MOESM1_ESM.pdf]

Coherent Mid-IR Supercontinuum Generation using Tapered Chalcogenide Step-Index Optical Fiber: Experiment and modelling

Than Singh Saini*, Tong Hoang Tuan, Takenobu Suzuki, and Yasutake Ohishi

Research Center for Advanced Photon Technology, Toyota Technological Institute, 2-12-1, Hisakata, Tempaku, Nagoya 468-8511, Japan

*E-mail: tsinghdph@gmail.com; tss@toyota-ti.ac.jp

Supplementary Material

1. METHOD OF ANALYSIS

1.1 Linear characteristic of the fiber

To simulate the tapered fiber structure, we calculated the effective mode indices of the fundamental mode by employing a commercially available software called 'COMSOL Multiphysics' which is based on full vectorial finite-element-method (FEM). In the simulation, the wavelength dependent refractive indices of the $AsSe_2$ and As_2S_5 chalcogenide materials were fitted using the Sellmeier equation given below [1]

$$n^2 = 1 + \sum_{n=1}^{5} \frac{A_n \lambda^2}{\lambda^2 - a_n^2} \qquad (1)$$

The numerical values of the Sellmeier coefficients are provided in the following Table-1.

Table-1: The Sellmeier coefficients

Selmeier coefficients	$AsSe_2$	As_2S_5
A_1	3.432139	1.223071
A_2	2.863671	2.805610
A_3	6.222296	4.600042
A_4	135.755231	0.379177
A_5	49.778749	13.561104
a_1	0.166298	0.367210
a_2	0.418493	0.114717
a_3	400.397723	151.604763
a_4	486.527641	27.912821
a_5	888.798916	710.164482

The group velocity dispersion contributes an important role in the broadening of supercontinuum spectrum. Group velocity dispersion determines the degree to which diverse spectral components of the ultra-short laser pulse propagate at different phase velocities in the fiber. The group velocity dispersion connected with the wavelength dependent effective mode indices of the propagating mode as the following relation [2]

$$D(\lambda) = -\frac{\lambda}{c} \frac{\partial^2 Re(n_{eff})}{\partial \lambda^2} \qquad (2)$$

where, c is the speed of the light in free space, $Re(n_{eff})$ represents the real part of effective mode indices.

The effective-mode-area of propagating mode is calculated by the following equation [2]

$$A_{eff} = \frac{\left(\iint_{-\infty}^{\infty} |E|^2 \, dx \, dy\right)^2}{\left(\iint_{-\infty}^{\infty} |E|^4 dx \, dy\right)} \qquad (3)$$

where, E represents the amplitude of the electric field.

1.2 Nonlinear characteristic of the fiber

In the simulation, an initial input pulse, a hyperbolic secant pulse is considered in the simulation which is given below

$$\mathrm{A}(0, \mathrm{T}) = \sqrt{\mathrm{P_0}} \, \mathrm{sech} \frac{\mathrm{T}}{\mathrm{T_0}} \qquad (4)$$

where A indicates an envelope of pulse, P_0 represents the peak power of pulse, $T_0 = T_{FWHM}/1.7627$ (T_{FWHM} represents the full-width-at-half-maxima) for a hyperbolic secant pulse, and T is the co-moving frame at the group velocity of the pulse envelope.

The supercontinuum spectrum generated in the fiber was simulated using the following generalized nonlinear Schrodinger equation [3]

$$\frac{\partial \tilde{A}'}{\partial z} = i\bar{\gamma}(\omega) \exp\left(-\hat{L}(\omega) z\right) \mathcal{F} \left\{ \bar{A}(z, T) \int_{-\infty}^{\infty} R(T') |\bar{A}(z, T - T')|^2 dT' \right\} \qquad (5)$$

where $\tilde{A}'$ represents the envelope of an output pulse in the frequency domain which is related to the envelope of the pulse in time domain by the following equation

$$\bar{A}(z, T) = \mathcal{F}^{-1} \left\{ \frac{\tilde{A}(z, \omega)}{A_{eff}^{\frac{1}{4}}(\omega)} \right\} \qquad (6)$$

where $\mathcal{F}^{-1}$ represents the inverse Fourier transform, z shows the propagation distance, and A_{eff} indicates the effective-mode-area of the mode propagating in the fiber.

The Eq. (5) was solved by employing the adaptive step size method with the fourth-order-Runge-Kutta algorithm [3].

$\bar{\gamma}(\omega)$ indicates the frequency dependent nonlinear coefficient and given by the following equation

$$\bar{\gamma}(\omega) = \frac{n_2 n_0 \omega}{c n_{eff}(\omega) A_{eff}^{1/4}(\omega)} \qquad (7)$$

where n_2 is the nonlinear refractive index ($n_2 = 2.3\times10^{-17}$ m^2/W for $AsSe_2$ based chalcogenide glass [4]), n_0 represents the linear refractive index of the glass at the wavelengths used to determine n_2, c indicates the velocity of the light in the vacuum, and n_{eff} is the effective refractive index of the propagating mode in the fiber.

The change of the variables is given by the relation

$$\tilde{A}'(z, \omega) = \tilde{A}(z, \omega) \exp(-\hat{L}(\omega) z) \qquad (8)$$

where $\hat{L}(\omega)$ indicates the linear operator which is given below

$$\hat{L}(w) = i(\beta(\omega) - \beta(\omega_0) - \beta_1(\omega_0)[\omega - \omega_0]) - \frac{\alpha(\omega)}{2} \qquad (9)$$

where β represents the propagation constant, β_1 is the reciprocal of group velocity of the envelope, ω_0 depicts the reference frequency, and α indicates the losses in the fiber. In the numerical simulations, both the material and confinement losses of the fiber have been included.

The Raman response function is calculated using the following relation

$$R(t) = (1 - f_R)\delta(t) + f_R \frac{\tau_1^2 + \tau_2^2}{\tau_1 \tau_2^2} \exp\left(-\frac{t}{\tau_2}\right) \sin\left(\frac{t}{\tau_1}\right) \mathrm{H(t)} \quad (10)$$

where f_R represents the fractional contribution of the Raman response, τ_1 is the Raman period, τ_2 provides the damping time of the network of vibrating atoms, and H(t) represents the Heaviside step function (H(t)=0 for t<0 & H(t)=1 fort>0). In the simulation, we have taken the fractional contribution f_R = 0.148, Raman period, τ_1=23 fs, and the life time, τ_2=164.5 fs [4].

1.3 Coherence characteristic of the generated supercontinuum spectrum

The coherence characteristic of the generated supercontinuum spectrum is affected by the existence of the quantum noise of the pulse. We used one-photon-per-mode semi-classical theory to model the noise of the input pulse [5]. The complex degree of coherence was used to consider the deficit in the coherence characteristic of the spectrum due to the spectral phase instability at each wavelength. The relation for the complex degree of coherence is as follows [6]

$$\left|g_{12}^{(1)}(\lambda, t_1 - t_2)\right| = \left|\frac{\langle E_1^*(\lambda, t_1) E_2(\lambda, t_2)\rangle}{\sqrt{\langle |E_1(\lambda, t_1)|^2\rangle} \sqrt{\langle |E_2(\lambda, t_2)|^2\rangle}}\right| \qquad (11)$$

where E_1 and E_2 are the amplitudes of the electric field for two successive generated spectra. To focus on the wavelength dependence of the coherence, in the simulation study t_1-t_2=0 was taken.

Reference

[1] K. Nagasaka, L. Liu, T. H. Tuan, T. Cheng, M. Matsumoto, H. Tezuka, T. Suzuki, and Y. Ohishi, "Numerical investigation of highly coherent midinfrared supercontinuum generation in chalcogenide double-clad fiber,"Opt. Fiber Technol., 36, 82–91, (2017).

[2] G. P. Agrawal, "Nonlinear Fiber Optics," 5th ed., Elsevier Academic Press, 2013.

[3] J. C. Travers, M. H. Fosz, and J. M. Dudley, Supercontinuum generation in optical fibers, J. M. Dudley and J. R. Taylor, eds. (Cambridge University Press, New York, 2010), pp.32 - 51.

[4] L. Liu, T. Cheng, K. Nagasaka, H. Tong, G. Qin, T. Suzuki, and Y. Ohishi, "Coherent mid-infrared supercontinuum generation in all-solid chalcogenide microstructured fibers with all-normal dispersion," Opt. Lett. 41(2), 392 – 395 (2016).

[5] M. H. Frosz, Validation of input-noise model for simulations of supercontinuum generation and rogue waves, Opt. Express 18, 14778 - 14787 (2010).

[6] J. M. Dudley, G. Genty, and S. Coen, Supercontinuum generation in photonic crystal fiber, Rev. Mod. Phys. 78(4), 1135–1184 (2006).
